# Short-term effects of dietary selenium on lactating sows to improve litter performance, milk composition and tissue selenium retention in piglets

**DOI:** 10.5713/ab.22.0425

**Published:** 2023-02-26

**Authors:** Xing Hao Jin, Hong Jun Kim, Cheon Soo Kim, Yoo Yong Kim

**Affiliations:** 1 Department of Agricultural Biotechnology and Research Institute of Agriculture and Life Science, Seoul National University, Seoul 08826, Korea

**Keywords:** Lactating Sows, Litter Performance, Milk Composition, Selenium

## Abstract

**Objective:**

This study was conducted to evaluate the short-term effects of dietary selenium supplementation on lactating sows on the physiological response, litter performance, milk composition, and tissue selenium retention in piglets when selenium was provided by different sources and at different levels in a lactation diet.

**Methods:**

A total of 48 multiparous sows (Yorkshire×Landrace) with average body weight, backfat thickness, and parity were assigned to one of the four treatments with 12 sows per treatment using a 2×2 factorial arrangement in a completely randomized design. Inorganic or organic Se sources were added to the diet at 0.30 ppm and 0.50 ppm Se. Treatments were as follows: i) IS30, basal diet + inorganic Se 0.30 ppm; ii) IS50, basal diet + inorganic Se 0.50 ppm; iii) OS30, basal diet + organic Se 0.30 ppm; and iv) OS50: basal diet + organic Se 0.50 ppm.

**Results:**

At Day 21 of lactation, a high tendency of litter weight (p = 0.08) and litter weight gain (p = 0.09) were observed when sows were fed an organic Se source. The milk Se concentration in the organic Se treatment was higher than that in the inorganic Se treatment at Day 21 of lactation (p<0.05). The serum Se concentrations of sows and piglets at Day 21 of lactation were significantly higher when lactating sows were fed organic Se instead of inorganic Se (p<0.01). During the suckling period, the kidney and muscle Se concentrations of piglets at Day 21 of lactation were significantly higher when the sow dietary Se source was organic (p<0.05). Liver Se concentrations were affected by Se source and level (p<0.05). This also resulted in an interaction response at 21 days of lactation (p<0.05).

**Conclusion:**

The supplementation of dietary organic Se in a lactating diet could improve sow feed consumption, piglet performance, milk Se level, and the Se status of sows and piglets.

## INTRODUCTION

Selenium (Se) is a critical trace mineral for animal growth and health [1]. Se sources added to the animal diet can be divided into two forms: inorganic and organic. Based on the respective characteristics of the two forms of Se, organic Se has a higher rate of absorption, tissue deposition, antioxidant bioavailability, and lower toxicities [2]. Thus, the application of dietary organic Se in swine feed has received more attention in recent years [3]. Many studies have indicated that the classifications of organic Se are chemosynthetic selenomethionine and Se yeast produced by the microbe yeast [4]. Therefore, organic Se may be more effective for Se addition than inorganic Se. Zhan et al [5] reported that organic Se has excellent potential in depositing Se in tissues and enhancing the antioxidant status.

The litter performance of piglets from birth to weaning is greatly affected by maternal nutrient levels and milk composition [6]. The Se status of sows and piglets was improved by the sow’s body Se reserves, dietary Se level, and source of Se [7,8]. Many reports have indicated that feeding a fortified organic Se diet could improve the amount of Se transferred from sows to newborn piglets [3,9]. Therefore, organic Se sources may be superior to inorganic Se sources, and maternal fortified Se intake may have a positive effect on the growth and metabolism of piglets [10]. However, the extent of Se deposition in offspring was inconsistent for the different Se sources and levels only during the lactation period [11].

In the current study, the effect of Se source and level during lactation on litter performance, serum Se content of sows and piglets, and milk composition in sows were examined. We hypothesized that the short-term effect of maternal Se supplementation could improve the sow performance, litter growth, and Se status of sows and piglets.

## MATERIALS AND METHODS

### Animals

All experimental procedures involving animals were conducted following the Animal Experimental Guidelines provided by the Seoul National University Institutional Animal Care and Use Committee (SNUIACUC; SNU-201112–3).

A total of 48 F1 multiparous sows (Yorkshire×Landrace) with an average body weight (BW) of 255.61 kg, an average backfat (BF) thickness of 25.01 mm, and an average parity of 4.6 were allotted to one of four treatments considering BW, BF thickness, and parity in a completely randomized design (CRD) after mating. All sows took two artificial insemination services according to the estrus cycle after weaning and were pregnancy checked at Day 35 of gestation by an ultrasound scanner (Donjin BLS, Gwangju, Korea).

### Experimental design and diet

The experiment was a 2×2 factorial arrangement in a CRD. Inorganic (sodium selenite) or organic (Se-enriched yeast) Se sources were added to the diet at 0.30 or 0.50 ppm Se. The experimental selenium sources added to the basal diet were sodium selenite (total Se 1,000 ppm, inorganic Se) and selenium yeast (total Se 1,000 ppm, organic Se; Sel-Plex, Alltech, South Brookings, SD, USA). The analyzed selenium (Se) concentrations were as follows: i) IS30, 0.42 ppm; ii) IS50, 0.62 ppm; iii) OS30, 0.45 ppm; and iv) OS50, 0.63 ppm. All experimental lactation diets were formulated to contain 3,300 kcal of ME/kg, 13.43% crude protein, 0.96% total lysine, 0.26% total methionine, 0.65% total threonine, 0.76% calcium and 0.65% total phosphorus. All other nutrients were formulated to meet or exceed the NRC [12] requirements. Table 1 shows the formulas and chemical compositions of the experimental diet.

### Animal management

A total of 48 pregnant sows were washed and moved into farrowing crates (2.40×1.80 m^2^) on Day 110 of gestation. The gestation diet was decreased gradually 0.2 kg per day for 5 days before farrowing. Delivery inducer was not used during farrowing, and all sows were assisted when dystocia occurred. After farrowing, the experimental lactation diet was increased gradually from 1.0 kg/d until 5 d postpartum and then provided *ad libitum* during the lactation period. The room temperature of the lactating barn was kept at 28°C±2°C, and the baby house under a heating lamp was kept at 32°C±2°C. The air condition of the lactating barn was regulated automatically by the ventilation system and air conditioner. After weaning, the sows were moved to a breeding barn for the next estrus cycle.

After farrowing, piglets were cross-fostered within treatment until 24 h postpartum to balance the suckling intensity of sows with equalization of litter size and thus to minimize any effect of initial litter size potentially affecting litter growth. Cutting of the umbilical cord and tail and castration were conducted 3 days after birth, and piglets were injected with 150 ppm Fe-dextran (Gleptosil, Alstoe, York, UK). None of the piglets were fed creep feed during the whole lactation period. Weaning was performed at approximately 25±3 d.

### Performance of the sow

The live BW and BF thickness of sows were measured at 24 h postpartum and 21 days of lactation, respectively. The BW of the sow was measured by an electric scale (CAS Co. Ltd., Yangju, Korea), and BF thickness was measured at the P2 position (mean value from both sides of the last rib and 65 mm away from the backbone) by an Ultrasound device (Lean Meter; Renco Corp., Minneapolis, MN, USA). Daily feed wastage was recorded during lactation, and lactation feed intake was estimated over 21 days to identify physiological effects on sows. The weaning to estrus interval (WEI) of sows was measured after weaning as one of the important parameters for evaluating reproductive performance.

### Litter performance

After farrowing, the number of piglets was recorded, and the BW of live piglets was measured by an electric scale (CAS CO. Ltd., Korea). When measuring the BW of piglets, ear notching was practiced for the experiment. After ear notching, cross-fostering of the piglets within the same treatment was performed until 24 h postpartum to equalize litter size. The number and BW of piglets were measured again at Day 21 of lactation to calculate litter weight, piglet weight, and weight gain.

### Blood samples

Blood samples (n = 6 for each treatment) were collected from the jugular vein of sows using 10 mL disposable syringes at 24 h postpartum and 21 days of lactation. Additionally, blood samples (n = 12) were collected from the anterior vena cava of piglets using 3 mL disposable syringes at 24 h postpartum and 21 days of lactation. The initial values were obtained randomly (n = 4 for sows and piglets) from the experimental animals after completed the experiment arrangement. All blood samples were moved to serum tubes (SSTTMII Advance; BD Vacutainer, Becton Dickinson, Plymouth, UK) and ethylenediaminetetraacetic acid tubes (BD Vacutainer K_2_E; Becton Dickinson, UK). Individual samples were centrifuged at 3,000 rpm and 4°C for 15 minutes (Eppendorf centrifuge 5810R; Hamburg, Germany), and the supernatant was separated into a microtube (Axygen, Union City, CA, USA) and stored at −20°C until analysis.

### Milk composition

Colostrum (n = 4 for random in sows) and milk samples (n = 6 for each treatment) were taken from functional mammary glands of each sow at 24 hours post-farrowing and on Day 21 of lactation, respectively. 5 mL of oxytocin (Komi oxytocin inj.; Komipharm International Co., Ltd., Siheung, Korea) was injected into the blood vessels of the sow’s ear. Colostrum and milk were collected in 50 mL conical tubes (SPL Life Sciences Co., Ltd., Pocheon, Korea) from the first and second teats. After collection, samples were stored in a freezer (−20°C) until further analysis. Proximate analysis of colostrum and milk was conducted using Milkoscan FT120 (FOSSElectric, Hillerød, Denmark).

### Tissue samples

Piglets were killed, and samples from the liver, kidney, and muscle were taken at 24 h postpartum (n = for random in piglets) and on 21 days of lactation (n = 4 for each treatment). Muscle samples were taken from the area of the muscle located between the 5th and 6th ribs. Piglets were humanely euthanized through CO_2_ inhalation and exsanguination. Individual samples were stored at −20°C until further analysis.

### Se analytical methods

Colostrum, milk, serum and tissues were analyzed according to the fluorometric method outlined by AOAC [13]. After the wet ashing of samples with nitric acid and perchloric acid at 160°C for approximately 2.5 to 3 h and the reduction with 6 mol/L hydrochloric acid, selenium was determined by 2,3-diamino-naphthalene fluorescence reaction.

### Statistical analysis

All of the collected data were subjected to least squares mean comparisons and evaluated with the general linear model (GLM) procedure of SAS [14]. Individual sows and their litters were used as the experimental unit The four treatments had organic or inorganic Se at 0.30 and 0.50 ppm and were analyzed as a 2×2 factorial, with the main effects and interactions evaluated. Differences among means were declared significant at p<0.05 and highly significant at p<0.01, and the determination of tendency for all analyses was p≥0.05 and p<0.10.

## RESULTS

### Performance of the sow

The short-term effects of dietary selenium source and level on BW, BF thickness, feed intake and WEI of lactating sows are shown in Table 2. Sow BW and weight changes were not affected by Se source or level. The changes in BF thickness tended to decrease when organic Se was added to the lactating sow diet (p = 0.06). Moreover, sows offered organic Se in the lactating diet showed a tendency towards higher feed intake than sows offered inorganic Se (p = 0.07).

### Litter performance

The effects of Se source and level on the litter performance of sows are shown in Table 3. At Day 21 of lactation, a high tendency of litter weight (p = 0.08) and litter weight gain (p = 0.09) was observed when sows were fed an organic Se source. However, the BW and weight gain of piglets were not affected by Se source or level.

### Milk composition

The effects of dietary Se sources and levels on the chemical composition of colostrum and milk in sows are shown in Table 4. In the present study, at Day 21 of lactation, the milk Se concentration in the organic Se treatment was higher than that in the inorganic Se treatment (p<0.05).

### Serum selenium concentration in lactating sows and piglets

The effects of dietary selenium source and level on serum Se concentration in sows and piglets are shown in Table 5. The Se concentrations of sows and piglets at Day 21 of lactation were significantly higher when lactating sows were fed organic Se instead of inorganic Se (p<0.01; p<0.01, respectively). According to the dietary Se level, no significant differences were observed.

### Tissue selenium concentration in piglets

The effects of selenium source and level on serum Se concentration in piglets are shown in Table 6. During the suckling period, the kidney and muscle Se concentrations of piglets at Day 21 of lactation were significantly higher when the sow dietary Se source was organic (p<0.05; p<0.05, respectively). Liver Se concentrations were affected by Se source and level (p<0.01, respectively). This also resulted in an interaction response at 21 days of lactation (p<0.05).

## DISCUSSION

Many previous studies have mainly focused on late gestation or transition [15,16]. In this study, the experimental period only concentrated on the lactation period, and short-term effects of Se source and level could be seen. Some studies reported that different Se sources and levels in a sow’s diet did not affect sow BW or their changes during the lactation period [17,18]. The current study showed that there were no significant differences in BW and BW changes by Se sources or levels, which matched previous studies. Backfat thickness is a significant parameter to evaluate fat storage in sows, and the change in BF thickness had a high correlation with sow BW loss and feed intake during the lactation period [19]. Mahan [20] reported that short-term feeding of either Se source at 0.15 or 0.30 ppm Se did not affect BF changes and feed consumption in lactating sows. Mahan and Peter [21] also reported that there were no significant improvements in sow BF thickness and feed intake during the lactation period when sows were fed different Se sources (organic Se or inorganic Se) and levels (0.15 or 0.30 ppm). However, in this study, BF change tended to decrease, and feed intake showed a higher tendency in both Se sources at the two levels. The reduction in sow BF thickness was also associated with low feed consumption [22]. Moreover, in the present study, the loss of sow BW was numerically lower when lactating sows were fed organic Se. The reason for the high tendency in feed consumption observed in organic Se could be related to the smell of the organic Se source (Se yeast). As smell is an initial attractant to feed, the sows fed Se yeast may consume more feed due to an appealing smell perception; a similar result was shown by Falk et al [23]. According to the performance of sows, including BW change and feed consumption, this study also indicated that sows fed organic Se sources could reduce BF loss to maintain a balance of anabolism and catabolism in the sow body pool and thus improve sow longevity. In addition, Se source and level did not affect WEI in the present study because BW loss and BF change were at normal levels during the lactation period.

Many studies have stated that maternal organic Se intake showed little effect on improving the growth performance of piglets during lactation [24,25]. Recently, Zhang et al [26] reported that litter BW and litter weight gain were potentially improved when the organic Se source was supplemented during lactation. Additionally, Mou et al [18] also showed that a significant improvement in the litter weight of piglets was observed as dietary organic Se supplementation to sow diets. In the present study, litter weight and litter weight gain tended to be higher when organic Se was added to the lactation diet, which was in agreement with previous studies. However, piglets’ BW and weight gain were not affected by Se source and level in either, which also suggested that adding dietary Se to the lactation diet could enhance milk yield to offer sufficient milk to the growth of piglets with low birth weight and could maintain high litter uniformity during lactation. The maternal nutrient level will improve the composition of both colostrum and milk, subsequently leading to a better performance of their progeny [26]. Falk et al [10] also reported that the improvement of growth performance and survival rate of piglets were seen as milk Se concentration increased. Therefore, an improvement in piglet performance had a highly significant correlation between litter performance and milk Se concentration, which was consistent with the results regarding milk composition as shown in Table 4.

Milk composition is more affected by maternal nutrition in the lactation period [27]. When the organic form of Se is used in a sow’s diet, the Se concentration in milk is higher than when the inorganic form is used [21,24]. Kelly and Power [28] reported that absorbed organic Se in the animal body had high efficiency to incorporate into milk in milk yield. This mechanism explains why the milk Se concentration was higher as the organic Se source was provided in the current study. Mahan [20] reported that milk Se content increased as dietary Se levels (0 ppm, 0.15 ppm, 0.30 ppm) increased when sows were fed organic Se or inorganic Se from 6 days prepartum to parturition. Surai [3] also stated that sow milk Se levels increased as the dietary Se level increased. Our data showed that there was no greater increase in milk Se contents as Se levels increased up to 0.5 ppm. It may be demonstrated that dietary Se levels did not affect milk Se content during the short-term lactation period around one reproductive cycle. Moreover, a report by Jin et al [29] also proved that despite Se source, dietary Se level could affect milk Se concentration linearly when sows were fed different Se levels (0 ppm, 0.3 ppm, 0.5 ppm) during gestation (long-term period).

During late gestation and lactation, the serum Se content of sows was higher when organic Se was supplied compared with inorganic Se offered [17,25]. Mahan and Kim [30] and Mahan and Peter [21] also reported that serum Se levels increased as dietary Se levels increased and when organic Se was fed compared with inorganic Se. In general, milk production mainly provides all nutrients to newborn piglets [31], and the Se status of piglets is improved by maternal Se source and level [32]. Mahan and Peter [21] found that piglet Se concentrations were improved when sows were fed different Se sources, especially organic forms. Mahan [21] also reported that the Se content of piglets in serum at birth and weaning age could be improved linearly with increasing levels of Se. Based on the above, the changes in serum Se of sows and piglets in the present study were similar to those in previous studies. However, in the present study, dietary Se levels did not affect the Se status of sows and piglets. This result means that the serum Se content of sows and piglets could be increased by maternal organic Se, regardless of an increase in Se levels in different Se forms in the lactating diets.

In general, there are great differences in Se concentrations among pig tissues, including kidney, liver and muscle tissues [33]. Seboussi et al [34] reported that selenium deposition in tissues gradually increases in the order of muscle, liver, and kidney. Mahan and Peters [21] reported that providing a fortified organic Se diet during gestation increased the Se content in sow and piglet tissues. Kim and Mahan [35] stated that the Se concentration in each of the tissues (kidney, liver, and muscle) increased as the dietary Se level increased, and the increase was greater when the organic Se source was fed to gestating sows. However, in the present study, organic Se treatments resulted in significant improvement in tissue Se concentrations in piglets, and there was no difference among dietary Se levels in the lactating sow. Zhan et al [15] reported that maternal organic Se intake during lactation significantly increased the Se content in the organs of piglets. This result indicated that the Se concentration in piglet tissue was affected only by the maternal Se source rather than the Se level in lactating sows, and dietary organic Se could improve the Se status of piglet tissues.

## CONCLUSION

The addition of organic Se in a lactating diet tended to improve sow feed consumption and piglet performance, and induce higher milk Se content than the addition of inorganic Se in sows. In addition, organic Se enhanced Se status of sows and piglets. However, the addition of high Se level to the feed of lactating sows did not have any positive effects. Therefore, dietary organic Se source to the feed of sows was beneficial in lactating period. Further research should be focused on the influence of mixed Se sources on sow performance, piglet performance, and the Se status of sows and piglets.

## Figures and Tables

**Table 1:** Formula and chemical compositions of lactation diet^1)^

Items	Lactation diet^2)^
Ingredients (%)	
Corn	73.16
Soybean meal, 48% CP	16.80
Wheat bran	3.32
Tallow	1.99
L-lysine HCl (50%)	0.63
DL-methionine (99%)	0.04
Threonine (98.5%)	0.17
Dicalcium phosphate	2.05
Limestone	1.14
Vit. Mix^3)^	0.10
Min.Mix^4)^	0.10
Choline chloride-50%	0.10
Salt	0.40
Sum	100.00
Chemical composition^5)^	
ME (kcal/kg)	3,300.00
Crude protein (%)	13.43
Lysine (%)	0.96
Methionine (%)	0.26
Threonine (%)	0.65
Ca (%)	0.75
Total P	0.65

ME, metabolizable energy.

1) Sodium selenite and Se yeast contained 1,000 ppm total selenium and were added to the basal diet to achieve the appropriate treatment levels.

2) Formulated to % Lys, % Met, % Thre, % Ca, and % P (total).

3) Provided the following per kilogram of lactation diet: vitamin A, 8,000 IU; vitamin D_3_, 1,600 IU; vitamin E, 32 IU; d-biotin, 64 g; riboflavin, 3.2 mg; calcium pantothenic acid, 8 mg; niacin, 16 mg; vitamin B_12_, 12 g; vitamin K, 2.4 mg.

4) Provided the following per kilogram of lactation diet: I, 0.3 mg; Mn, 24.8 mg; CuSO4, 54.1 mg; Fe, 127.3 mg; Zn, 84.7 mg; Co, 0.3 mg.

5) Calculated values.

**Table 2:** Effects of selenium source and dietary level in lactation diet on body weight, backfat thickness, weaning to estrus interval and lactation feed intake of sows

Items	Inorganic Se (ppm)		Organic Se (ppm)		SEM	p-value		
		
	0.3	0.5	0.3	0.5		Source (S)	Level (L)	S×L
No. of sows farrowed	12	12	12	12				
Body weight (kg)								
24 h postpartum	254.63	256.97	256.40	254.90	4.431	0.98	0.84	0.96
21 d of lactation	244.63	242.50	246.50	248.00	4.742	0.88	0.98	0.92
Change (0 to 21 d)	−10.00	−14.47	−9.90	−6.90	1.564	0.59	0.52	0.82
Backfat thickness (mm)								
24 h postpartum	25.50	25.17	24.37	25.00	0.472	0.14	0.19	0.51
21 d of lactation	22.13	22.00	21.83	22.83	0.322	0.69	0.42	0.53
BF change (0 to 21 d)	−3.07	−3.06	−2.17	−2.53	0.191	0.06	0.62	0.60
Lactation feed intake (kg/d)	6.07	6.03	6.22	6.20	0.423	0.07	0.76	0.87
WEI (d)	4.75	4.61	4.62	4.50	0.071	0.69	0.71	0.15

SEM, standard error of the mean; WEI, weaning to estrus interval.

**Table 3:** Effects of selenium source and dietary level in lactation diet on litter performance

Items	Inorganic Se (ppm)		Organic Se (ppm)		SEM	p-value		
		
	0.3	0.5	0.3	0.5		Source (S)	Level (L)	S×L
No. of sows	12	12	12	12				
No. of piglets								
Total born	13.00	13.50	13.00	13.00	0.222	0.68	0.68	0.68
Born alive	11.33	11.25	11.50	12.00	0.203	0.64	0.93	0.92
After cross-foster^1)^	11.33	11.25	11.40	11.50	0.221	0.34	0.54	0.93
21 d of lactation	10.67	10.35	11.00	10.75	0.172	0.26	0.37	0.82
Litter weight (kg)								
Total litter weight	17.25	17.50	17.01	17.56	0.441	0.93	0.68	0.87
Litter birth weight	16.17	15.84	16.27	16.85	0.412	0.54	0.89	0.61
After cross-foster	16.17	15.84	16.37	16.85	0.414	0.54	0.97	0.63
21 d of lactation	60.08	56.95	65.90	65.41	1.933	0.08	0.73	0.64
Litter weight gain (0 to 21 d)	43.91	41.12	49.51	48.56	1.781	0.09	0.60	0.79
Piglet weight (kg)								
Piglet birth weight	1.42	1.43	1.43	1.47	0.052	0.86	0.85	0.83
After cross-foster	1.43	1.45	1.41	1.47	0.051	0.94	0.70	0.84
21 d of lactation	5.60	5.58	6.00	6.09	0.152	0.18	0.92	0.86
Piglet weight gain (0 to 21d)	4.17	4.13	4.59	4.62	0.143	0.14	0.97	0.91

SEM, standard error of the mean.

1) After cross-fostering day within 24 hours postpartum.

**Table 4:** Effects of selenium source and dietary level in lactation diet on chemical composition of milk of sows

Items	Inorganic Se (ppm)		Organic Se (ppm)		SEM	p-value		
		
	0.3	0.5	0.3	0.5		Source (S)	Level (L)	S×L
Selenium (ppm)								
Colostrum	0.063							
Milk (21 d)	0.059	0.060	0.085	0.130	0.009	0.01	0.11	0.13
Casein (%)								
Colostrum	8.23							
Milk (21 d)	3.86	3.84	3.83	3.79	0.042	0.691	0.796	0.924
Fat (%)								
Colostrum	5.79							
Milk (21 d)	6.28	6.20	6.15	6.39	0.192	0.93	0.82	0.63
Protein (%)								
Colostrum	12.15							
Milk (21 d)	4.70	4.68	4.67	4.65	0.062	0.79	0.87	0.99
Lactose (%)								
Colostrum	3.46							
Milk (21 d)	5.62	5.76	5.76	5.77	0.021	0.34	0.32	0.41
Total solid (%)								
Colostrum	23.95							
Milk (21 d)	18.52	18.24	18.06	18.59	0.215	0.87	0.72	0.27
Solid not fat (%)								
Colostrum	16.83							
Milk (21 d)	11.00	11.17	11.17	11.06	0.052	0.90	0.90	0.51

SEM, standard error of the mean.

**Table 5:** Effects of selenium source and dietary level in lactation diet on serum Se concentration of lactating sows and piglets

Items	Inorganic Se		Organic Se		SEM	p-value		
		
	0.3	0.5	0.3	0.5		Source (S)	Level (L)	S×L
Sow serum selenium (ppm)								
Initial		0.167						
21 d of lactation	0.203	0.201	0.258	0.277	0.039	0.01	0.16	0.20
Piglet serum selenium (ppm)								
Initial		0.100						
21 d of lactation	0.073	0.074	0.092	0.124	0.034	0.01	0.18	0.14

SEM, standard error of the mean.

**Table 6:** Effects of selenium source and dietary level in lactation diet on serum Se concentration of piglets

Items	Inorganic Se		Organic Se		SEM	p-value		
		
	0.3	0.5	0.3	0.5		Source (S)	Level (L)	S×L
No. of piglets	4	4	4	4				
Kidney Se (ppm)								
24 h postpartum		0.465						
21 d of lactation	0.444	0.453	0.675	0.772	0.063	0.01	0.22	0.19
Liver Se (ppm)								
24 h postpartum		0.285						
21 d of lactation	0.321	0.318	0.373	0.564	0.051	0.01	0.01	0.01
Muscle Se (ppm)								
24 h postpartum		0.011						
21 d of lactation	0.027	0.025	0.030	0.036	0.012	0.05	0.41	0.22

SEM, standard error of the mean.
